# Successful Treatment of* Bacillus cereus* Bacteremia in a Patient with Propionic Acidemia

**DOI:** 10.1155/2016/6380929

**Published:** 2016-04-19

**Authors:** Fatma Deniz Aygun, Fatih Aygun, Halit Cam

**Affiliations:** ^1^Department of Pediatric Infectious Diseases, Clinical Immunology and Allergy, Istanbul University, Cerrahpasa Medical Faculty, 34098 Istanbul, Turkey; ^2^Department of Pediatric Intensive Care Unit, Istanbul University, Cerrahpasa Medical Faculty, 34098 Istanbul, Turkey

## Abstract

*Bacillus cereus* can cause serious, life-threatening, systemic infections in immunocompromised patients. The ability of microorganism to form biofilm on biomedical devices can be responsible for catheter-related bloodstream infections. Other manifestations of severe disease are meningitis, endocarditis, osteomyelitis, and surgical and traumatic wound infections. The most common feature in true bacteremia caused by Bacillus is the presence of an intravascular catheter. Herein, we report a case of catheter-related bacteremia caused by* B. cereus* in a patient with propionic acidemia.

## 1. Introduction

*Bacillus cereus (B. cereus)* is an aerobic gram-positive, spore-forming, rod-shaped bacterium that is ubiquitous in the environment.* B. cereus* belongs to the* Bacillus* genus, along with* Bacillus anthracis*,* Bacillus thuringiensis*,* Bacillus mycoides*,* Bacillus pseudomycoides*,* Bacillus weihenstephanensis*, and* Bacillus toyonensis*. The members of the* Bacillus cereus* group have extremely similar properties and the autonomic differentiation system is not sufficient to determine the species of the genus. They are identified by differences in plasmid content, morphological structure, and pathogenicity.* Bacillus cereus* is the most common human pathogen of the group [1].

*Bacillus* spores are abundant in soil, fresh water, and hospital environment and even in normal gastrointestinal flora of prolonged hospitalized patients. It is commonly associated with toxin-mediated foodborne acute gastroenteritis, which is mostly self-limiting and benign [2, 3]. However, it can cause fatal systemic infections among neonates, immunocompromised patients, and intravenous drug users [2, 4]. Other manifestations of severe disease are meningitis, endocarditis, osteomyelitis, and surgical and traumatic wound infections, but they are rare and mainly limited to case reports [4, 5].* Bacillus cereus* is usually considered as contaminant when it is isolated from clinical specimens. The most common feature in true bacteremia caused by* Bacillus* species is the presence of an intravascular catheter [4]. Herein, we report a case of catheter-related bacteremia caused by* B. Cereus* following acute gastroenteritis in a patient with propionic acidemia.

## 2. Case Report

A 16-month-old male patient diagnosed with propionic acidemia was hospitalized for metabolic acidosis and hypoglycemia following acute gastroenteritis. Acute management of patient including high-caloric nutrition and correction of acidosis was done via central venous catheter and his metabolic status improved. At the third day, while he was kept in for rest of treatment and setting of diet, he developed fever, tachypnea, tachycardia, and hypotension and was transported to intensive care department with diagnosis of septic shock. Noradrenaline infusion was started for deep persistent hypotension in spite of three times of isotonic saline solution. Dobutamine was started for cardiac decompensation and he was intubated for respiratory insufficiency. Laboratory studies revealed total leukocyte count of 3700/mm^3^, neutrophil of 1800, platelet count of 33 000/mm^3^, C-reactive protein of 19.4 mg/dL, blood pH of 7.36, base excess of −11.2 mmol/L, HCO_3_ of 14.3 mmol/L, pCO_2_ of 25.7 mmHg, and lactate of 4.2. Serum ammonia was 71 mmol/L. Vancomycin, meropenem, and amikacin sulfate were started. Platelet and fresh frozen plasma transfusion were given for prolonged coagulation tests.* Bacillus cereus* was isolated in blood cultures obtained from the catheter and from a peripheral vein. The strain was sensitive to meropenem and amikacin. The patient's condition improved quickly with metabolic replacement therapy and antibiotic use for 14 days. He was discharged with outpatient control scheduling.

## 3. Discussion

*Bacillus cereus* is an opportunistic member of the the* Bacillus cereus* group, bearing several close phenotypic and genetic features with other* Bacillus* species. Three of them especially,* Bacillus anthracis*,* Bacillus thuringiensis*, and* Bacillus cereus*, have very similar chromosomal structure [6]. However, recently introduced genetic markers such as the BA5345 can be used as a chromosomal marker in routine identification of* B. anthracis* [7]. Also,* B. anthracis* contains two plasmids, pXO1 and pXO2, which encodes encapsulation and toxin production. The plasmids of* B. anthracis* determine the specificity of the bacteria [1]. Similarly,* B. thuringiensis* is indistinguishable from* B. cereus*, but* B. thuringiensis* is primarily an insect pathogen and detected by crystalline toxin inclusions during sporulation [5].

*Bacillus cereus* is saprophytic, disinfectant resistant environmental bacteria that can cause severe life-threatening systemic infections in immunocompromised patients. It is also an increasingly emerged cause of life-threatening infections in patients with hematologic malignancies and premature infants having invasive procedures, such as central catheterization and prolonged mechanical ventilation. The other high risk groups are intravenous drug users, neurosurgical patients with intraventricular shunts, and patients having penetrating trauma, intrathecal chemotherapy, and anaesthesia [8].

The possible source of bacteremia among these patients is usually central venous catheter, because the microorganism forms a biofilm on biomedical devices which can play a major role in attachment to catheters.* Bacillus* species are associated with catheter-related bloodstream infections, especially among patients with hematological malignancies.

To our knowledge, the case we report is the only patient with the diagnosis of propionic acidemia developing* Bacillus cereus* bacteremia. Patients with neutropenia, recurrent hospitalisation, and systemic corticosteroids usage have unfavorable outcome during bacteremia. The neutropenia is reported to be as high as 80% among patients with hematological malignancies having* Bacillus cereus* bacteremia and the resolution of neutropenia improves the prognosis. Bone marrow suppression and neutropenia have been reported in association with propionic acidemia but our case was not neutropenic.

The bacterium produces several exotoxins including cereolysin, lecithinase, phospholipase, and proteases that are responsible for tissue damage and progression of the infection. Patients may develop rapidly fulminant septic shock and coma during bacteremia. The rate of mortality of septic shock is reported to be 20% by Kato et al. [9].

The cause of mortality can be the resistance of* B. cereus* to penicillins and cephalosporins as a result of beta lactamase production [2]. Early administration of appropriate antibiotic treatment is important to prevent progression of the disease and the mortality. Vancomycin appears to be the most suitable treatment of choice for* B. cereus* bacteremia. However, carbapenem antibiotics are reported to be as effective as glycopeptide group [2, 5].

In conclusion, isolation of* Bacillus cereus* in the blood culture from a patient with chronic disease and septic shock should not be viewed routinely as a contaminant and should be considered as a potential pathogen. Early treatment with appropriate antibiotics should be started.
